# Stage at Diagnosis Following Delay to Interval Scans for Indeterminate Nodules in Lung Cancer Screening: An Observational Study Examining the Outcomes of CHEST Expert Panel Recommendations

**DOI:** 10.1016/j.chest.2023.10.033

**Published:** 2023-10-31

**Authors:** Andrew W. Creamer, Carolyn Horst, Jennifer L. Dickson, Sophie Tisi, Helen Hall, Priyam Verghese, Ruth Prendecki, Amyn Bhamani, Chuen R. Khaw, John McCabe, Tanita Limani, Kylie Gyertson, Anne-Marie Hacker, J. Teague, L. Farrelly, Shrinkhala Dawadi, Allan Hackshaw, Anand Devaraj, Arjun Nair, Sam M. Janes

**Affiliations:** a Lungs For Living, UCL Respiratory, University College London, London, England; b University College London Hospitals NHS Foundation Trust, London, England; c Cancer Research UK and UCL Cancer Trials Centre, University College London, London, England; d National Heart and Lung Institute, Imperial College, London, England; e Royal Brompton Hospital; and Harefield Hospital, Uxbridge, England

To the Editor:

The SARS-CoV-2 pandemic necessitated urgent adaptation of guidelines to ensure individuals were exposed to health care settings only when absolutely necessary. This included the follow-up of pulmonary nodules, especially in lung cancer screening participants. Although delaying imaging follow-up evaluation in this context seems prudent, such delays theoretically risk upstaging of early-stage lung cancers that manifest as nodules. To assist practitioners who facilitate lung cancer screening programs, the CHEST expert panel report recommended that screening should be paused and that, for indeterminate nodules that require surveillance, the time intervals could be relaxed by 3-6 months.^1^

Although the World Health Organization has declared an end to SARS-CoV-2 as a global health emergency,^2^ participant adherence to strict timing of interval surveillance scans has been shown to be suboptimal.^3^ However, at this time, little is known regarding the effect of such delays on stage at diagnosis.

The SUMMIT Study is a prospective observational cohort study that examines the delivery of a low-dose CT (LDCT) scan lung cancer screening service to a high-risk population and that validates a multicancer early detection blood test.^4^ Running from 2019-2023, the study was impacted by national lockdowns caused by the SARS-CoV-2 pandemic, which means that a proportion of participants had delays to interval scans for indeterminate nodules identified on baseline LDCT. The aim of this observational analysis was to examine whether the delay of interval scans for indeterminate nodules within the timescale proposed by the CHEST expert panel was associated with an increased proportion of cancers diagnosed at stage 2 or above.

## Patients and Methods

### The SUMMIT Study

Eligible participants were 55-77 years old who met the 2013 US Preventive Services Task Force-like screening criteria or had Prostate, Lung, Colorectal, and Ovarian screening model (PLC0_m2012_) risk of ≥ 1.3%. Images were read by thoracic radiologists who used computer-aided detection software (Veolity, version 1.4; MeVIS Medical Solutions) with semiautomated volumetry.

The SUMMIT nodule management protocol^5^ was based on British Thoracic Society (BTS) guidelines.^6^ Solid nodules of ≥ 80 mm^3^ and < 300 mm^3^ and part-solid nodules on baseline scan undergo interval surveillance LDCT at 3 months (nodule follow-up CT scan) with referral for definitive assessment by the multidisciplinary team if there is clear evidence of growth.^7^ Indeterminate consolidation (analogous to the indeterminate infectious or inflammatory findings where a clearly inflammatory cause cannot be determined defined by the Lung Imaging Reporting and Data System (Lung-RADS, version 2022^8^) also underwent nodule follow-up CT scan at 3 months.

Although nodule management was undertaken according to BTS guidelines,^6^ Lung-RADS categories^8^ were determined to facilitate comparison with screening programs that use the Lung-RADS protocol as a secondary analysis.

Participants were recruited from April 2019-May 2021; 12,961 participants underwent a baseline LDCT. In response to national lockdowns, study sites closed between March 25 and July 7, 2020, and January 8-March 1, 2021. Participants due for a nodule follow-up CT scan during a period of closure had these scheduled as soon as possible after the sites were reopened.

### Data Analysis

Time to nodule follow-up CT scan was determined in all study participants with an indeterminate baseline nodule. Stage was assigned according to the TNM staging, with pathologic stage used after surgical resection and clinical stage when no surgery had been performed.

Cancers diagnosed after further CT scans within SUMMIT or those diagnosed outside of the study were not included in this analysis. Time to nodule follow-up CT scan was analyzed as a categoric variable (88-120, 121-150, 151-180, and 181-364 days). Because the aim of the current study was to determine the impact of delaying nodule follow-up CT scans for indeterminate nodules rather than annual surveillance CT scans, the time-to-interval screening was capped at 364 days.

Differences in stage at diagnosis was analyzed by stage I vs stage > I. Fisher exact test was used to obtain *P* values for the proportion of participants diagnosed with stage I vs stage > I lung cancer in each time to nodule follow-up CT category. Relative risk was calculated relative to scans performed within the target timeframe of 88-120 days. All data analysis was performed with the use of R (version 4).

## Results

Of the 12,961 participants who had a baseline LDCT, 2,001 participants (15.4%) underwent a first nodule follow-up CT scan within the study between 88 and 364 days. Of the 2,001 participants, 1,292 procedures (64.6%) were performed within the target timeframe of 90 to 120 days; 493 procedures (24.6%) were performed between 4 and 6 months, and 216 procedures (10.8%) performed at > 6 months (Table 1).

After this, 160 of the 2,001 participants (8.0%) were referred to the multidisciplinary team for definitive assessment of growing nodules. A total of 87 participants received a diagnosis of lung cancer after this referral. Per Fisher exact test, neither the proportion of participants referred for definitive assessment nor the proportion of those referred who ultimately received a diagnosis of lung cancer changed significantly with time to nodule follow-up CT scan (Table 1).

**Table 1:** Stage at Diagnosis By Time to Nodule Follow-Up CT Scan: All Participants

Variable**s**	Time to Scan**, d**			
	88-120	121-150	151-180	181-364
Participants undergoing nodule follow-up CT scan, No.	1,292	259	234	216
Participants referred for multidisciplinary team assessment (% of participants undergoing nodule follow-up CT scan)	114 (8.98)	15 (5.79)	19 (8.12)	12 (5.56)
Total lung cancers diagnosed (% of participants referred for multidisciplinary team assessment)	59 (51.8)	10 (66.7)	12 (63.2)	6 (50)
*P* value	NA	.410	.458	1.0
Stage at diagnosis				
IA	43	9	10	2
IA1	7	2	2	1
IA2	32	6	6	0
IA3	4	1	2	1
IB	8	1	0	1
IIA	1	0	0	0
IIB	2	0	0	2
IIIA	4	0	2	0
IIIB	0	0	0	0
IIIC	1	0	0	0
IV	0	0	0	1
Cancers diagnosed at stage > 1, No. (%)	8 (13.6)	0 (0)	2 (16.7)	3 (50)
*P* value	NA	.592	.673	.056
Relative risk (95% CI)	NA	0.0	1.23 (0.30-5.08)	3.69 (1.32-10.30)
Cancers diagnosed at stage >1A1, No. (%)	52 (88.1)	8 (80)	10 (83.3)	5 (83.3)
*P* value	NA	.609	.643	.499
Relative risk (95% CI)	NA	0.91 (0.66-1.25)	0.95 (0.72-1.24)	0.95 (0.65-1.37)

NA = not applicable.

The overall risk of a participant receiving a diagnosis of lung cancer of stage > 1 after a nodule follow-up CT scan that was performed within the target timeframe of 88-120 days was 0.62% (95% CI, 0.27-1.22), 121 to 180 days was 0.41% (95% CI, 0.05-1.46; *P* = .736) and at > 180 days was 1.39% (95% CI, 0.03-4.01; *P* = .200). Table 1 presents the relative risk of a stage > 1 diagnosis by delays to nodule follow-up CT scan as compared to those performed in the target timeframe.

The proportion of cancers diagnosed at stage > I remained stable when nodule follow-up CT scan was performed between 3 and 6 months but rose once nodule follow-up CT scan was delayed beyond 6 months (≥ 181 days; relative risk, 3.69; 95% CI, 1.32-10.30; *P* = .056). Fisher exact test indicated no significant difference in the proportion of lung cancers diagnosed at stage > 1A1 after nodule follow-up CT scans performed between 88-120 days and 121 to 150 days (relative risk, 0.91 (*P* = .609), at 151-180 days (relative risk, 0.95; *P* = .643), or 181-365 days (relative risk, 0.95; *P* = .499).

The number of participants for whom nodule follow-up CT scans were performed at ≥ 181 days was small; hence, the number of cancer diagnoses was also low (n = 6). Therefore, there were insufficient cases to perform more detailed analysis (eg, stratification by initial nodule size/malignancy probability).

To allow extrapolation to screening programs with the use of the Lung-RADS protocol for management of findings,^8^ we analyzed our findings according to Lung-RADS categories at baseline LDCT (Table 2). We performed a secondary analysis limited to cancers diagnosed in participants with LungRADS categories 4A, 4B, 4X, or indeterminate consolidation (ie, follow-up CT scan was due at 3 months) (Table 3). We observed no significant difference in stage distribution when nodule follow-up CT scans were performed within the target timeframe (88-120 days) and 121-150 days (relative risk, 0; *P* = .579) and 151-180 days (relative risk, 1.51; *P* = .626), and a trend to a higher proportion of cancers being diagnosed at stage > I when nodule follow-up CT scans were delayed beyond 181 days (relative risk, 3.03; *P* = .168).

**Table 2:** Lung Imaging Reporting and Data System (Lung-RADS; version 2022) Categories of Participants at Baseline Low-Dose CT Scan

Lung-RADS Category at Baseline Low-Dose CT	Participants, No. (%)	Participants Diagnosed With Cancer After Referral at 3-mo Nodule Follow-Up CT Scan, No. (%)
Total	2,001 (100)	87 (4.3)
1	0 (0)	0 (0)
2	126 (6.3)	1 (0.8)
3	762 (38.1)	10 (1.3)
4A	594 (29.7)	30 (5.1)
4B	74 (3.7)	29 (39.2)
4X	10 (0.5)	1 (10)
Indeterminate consolidation	435 (21.7)	16 (3.7)

Lung-RADS = Lung Imaging Reporting and Data System.

**Table 3:** Stage at Diagnosis by Time to Nodule Follow-Up CT Scan in Participants Diagnosed With Lung Cancer Where Initial Lung Imaging Reporting and Data System (Lung-RADS; version 2022) Category Was 4A, 4B, 4X, or Indeterminate Consolidation

Variable**s**	Time to Scan, d			
	88-120	121-150	151-180	181-364
Total lung cancers diagnosed, No.	53	8	10	5
Stage at diagnosis, No.				
IA	39	7	8	2
IA1	7	1	0	1
IA2	28	5	6	0
IA3	4	1	2	1
IB	7	1	0	1
IIA	1	0	0	0
IIB	2	0	0	1
IIIA	3	0	2	0
IIIB	0	0	0	0
IIIC	1	0	0	0
IV	0	0	0	1
Cancers diagnosed at stage > I, No. (%)	7 (13.2)	0 (0)	2 (20)	2 (40)
*P* value	NA	.579	.626	.168
Relative risk (95% CI)	NA	0.0	1.51 (0.37-6.26)	3.03 (0.85-10.85)
Cancers diagnosed at stage > IAI, No. (%)	46 (86.8)	7 (87.5)	10 (100)	4 (80)
*P* value	NA	1.00	.585	.538
Relative risk (95% CI)	NA	1.01 (0.76-1.34)	1.15 (1.04-1.28)	0.92 (0.59-1.45)

*P* values were calculated with Fisher exact test, comparing the proportion of patients in each time-to-scan category with those scans performed in the target timeframe of 88-120 days. Cancer staging was determined according to TNM staging. Lung-RADS = Lung Imaging Reporting and Data System; NA = not applicable.

## Discussion

In a cohort of 2,001 participants who underwent interval surveillance (due at 3 months) for an indeterminate nodule at baseline LDCT, we found no change in the proportion of participants diagnosed with stage > I lung cancer when nodule follow-up CT scans were delayed from 3 to 6 months. We observed a higher proportion of cancers that were diagnosed at stage II or above when scans were delayed beyond 6 months, which suggests that delays beyond this timepoint risk disease progression becoming significant.

Although there is evidence that adherence to recommended follow-up for screen-detected findings is known to be suboptimal,^3^ there is little evidence for the consequences of such delays. Such research is challenging; first, most participants who undergo nodule follow-up CT scan will not have cancer, meaning large screened cohorts are required to obtain sufficient cancer numbers. Second, given that pathologic upstaging at surgery occurs in 17%-38% of cases,^9^^,^^10^ it is not possible to determine whether extended delays truly led to stage shift in individual cases. With the use of a retrospective review of baseline (noncontrast) LDCT examinations to assess for clinical stage shift, a recently published article in a US screening cohort^11^ found that clinical upstaging was observed more frequently following delayed nodule follow-up CT scans compared with cancers diagnosed following nodule follow-up CT scans performed at the target time, with a median delay in cases in which upstaging occurred of 131 days (after the target time). Although using different methodological approaches, our findings in nodule follow-up CT scans performed at > 180 days (equivalent to delays of > 90 days after target time) are concordant with those of Ahmed et al,^11^ with more prolonged delays to nodule follow-up CT scans increasing the risk of cancers being diagnosed at higher stage.

Our findings should not be interpreted as giving the “optimal” time to perform surveillance scans. There is already discrepancy between LungRADS, which recommends 3-month follow-up for higher risk and 6-month follow-up for lower risk nodules, and BTS^6^ and European position statement for lung cancer screening guidelines,^12^ which recommend a repeat LDCT at 3 months. Determining the optimal nodule follow-up CT scan interval must consider the risk of stage shift while ensuring enough time for true growth to be observed and for feasibility for participants and screening programs.

Our comparative analysis is limited by the fact that we were able to perform the vast majority of nodule follow-up CT scans within 6 months; therefore, only a small number of participants were scanned after an interval of > 180 days. Nevertheless, it is worth noting that the 2,001 individuals and 87 cancers included in this analysis are comparable with other entire screening cohorts.^13^ A further limitation is that this is observational data. Finally, although LungRADS categories were available for secondary analysis, our nodule management protocol used BTS guidelines.

In conclusion, in a cohort of 2,001 participants who underwent NFU CT for indeterminate nodules that were identified at baseline LDCT, we observed no difference in risk of lung cancer diagnosis at stage > 1 when nodule follow-up CT scans were delayed from 3-6 months, but we observed a higher proportion of cancers diagnosed at stage II or above when scans were delayed beyond 6 months. This finding provides support to the guidelines proposed by the CHEST expert panel^1^ that, during the SARS-CoV-2 pandemic at which time attending a CT scan in a health care setting poses a significant risk to a participants’ health, deferring interval surveillance for indeterminate nodules that were identified on baseline scans by up to 6 months is a justifiable approach, However, the increased risk of higher stage at diagnosis beyond 6 months should be recognized, with every effort made to ensure that indeterminate nodules undergo surveillance before this.

## Funding/Support

The SUMMIT study is funded by GRAIL through a research grant awarded to S. M. J. as principal investigator. S. M. J. was a Wellcome Trust Senior Fellow in Clinical Science (WT107963AIA); he is supported by CRUK programme grant EDDCPGM/100002, the Rosetrees Trust, the Roy Castle Lung Cancer foundation, the Garfield Weston Trust and UCLH Charitable Foundation.

## Financial/Nonfinancial Disclosures

The authors have reported to *CHEST* the following: A. W. C., C. H., J. L. D., S. T., H. H., P. V., R. P., A. B., and C. R. K. are all funded or part-funded through GRAIL as part of the SUMMIT Study. SUMMIT is sponsored and conducted by University College London and funded by GRAIL LLC through a research grant awarded to S. M. J. as principal investigator. S. M. J. is a paid Advisory Board member Astra-Zeneca, Bard1 Bioscience, Achilles Therapeutics, and Jansen; receives assistance for travel to meetings from Astra Zeneca, Takeda; receives grant income from GRAIL Inc, Owlstone; and receives share options from Optellum and BARD1 Lifescience. A. N. is part-funded through the UCLH Biomedical Research Centre. A. D. receives personal fees from Boehringer Ingelheim, Roche, Galacto Biotech, Galapagos, Brainomix and Vicore. A. H. receives consulting fees from Evidera and assistance for travel to meetings from GRAIL. None declared (J. M., T. L., K. G., A.-M. H., J. T., L. F., S. D.).
